# Supplementary figures and images for: Differential activation of mouse and human Panx1 channel variants
Source: PLoS One. 2023 Dec 15;18(12):e0295710. doi: 10.1371/journal.pone.0295710 (PMC10723736; doi:10.1371/journal.pone.0295710)

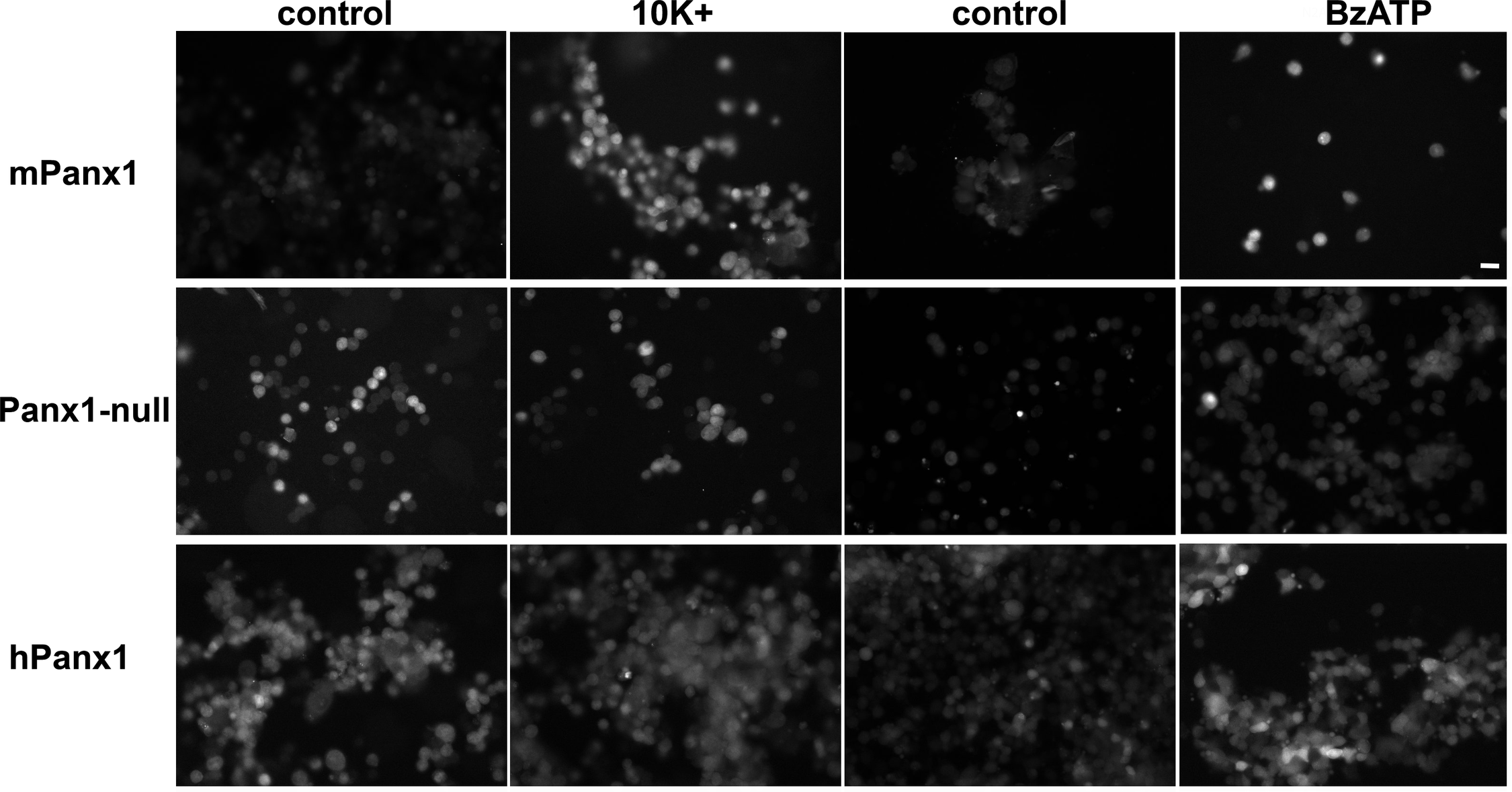

Supplement: S1 Fig — Images were acquired from 4% PFA fixed cells. Scale bar: 20 μm. (TIF) [file pone.0295710.s001.tif]

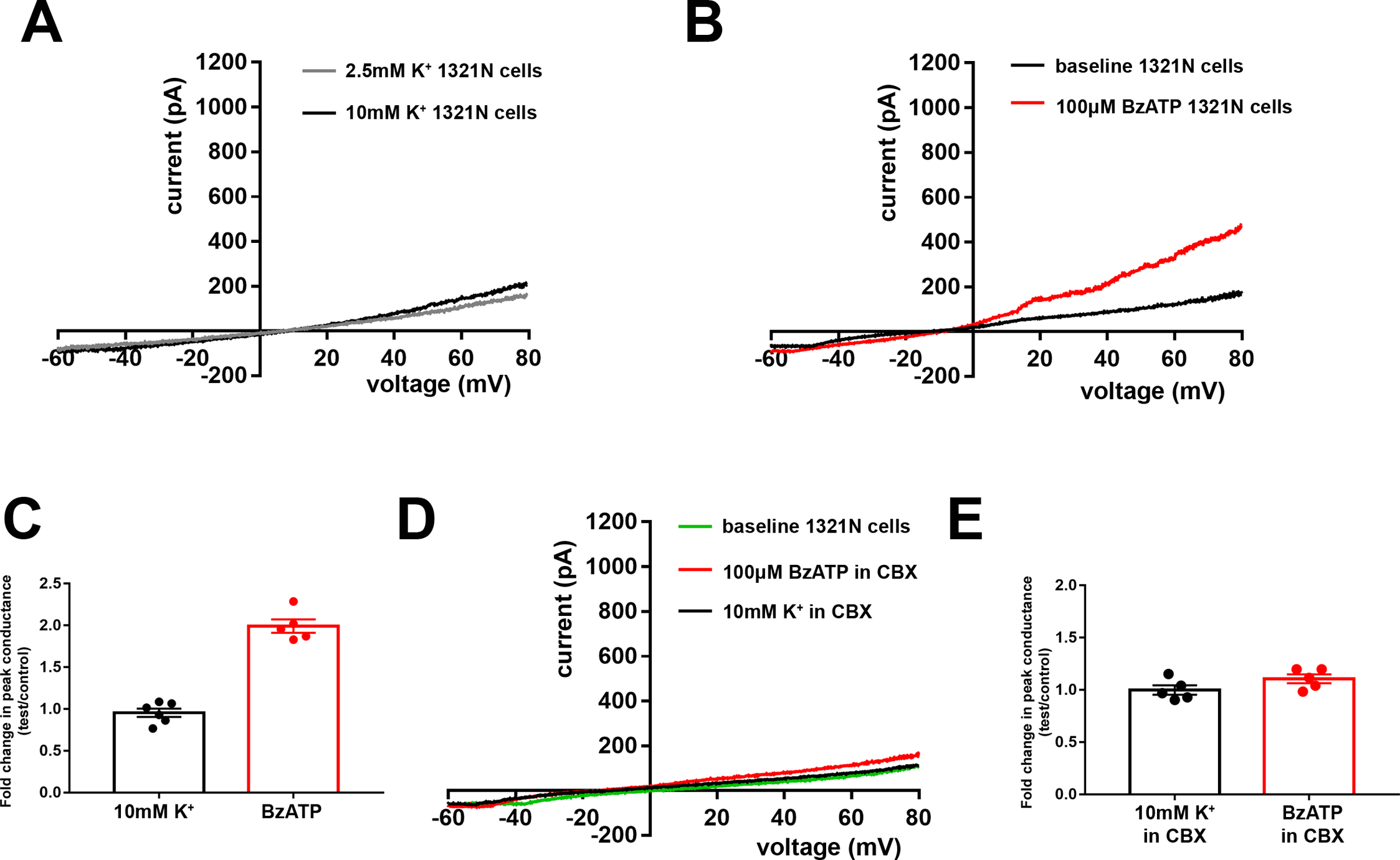

Supplement: S2 Fig — (A-B) Examples of hPanx1 currents recorded from 1321N1 human astrocytoma cell line expressing rP2X7 receptor exposed to solutions containing 2.5 and 10 mM K+ and to 100 μM BzATP. (C) Fold changes in peak conductance measured from 1321N1 cells exposed to high potassium (n = 6) and BzATP (n = 5). Note that elevated extracellular K+ concentration did not lead to increase in Panx1 currents above baseline, while exposure to BzATP caused a 2 fold increase in peak conductance. (D-E) Examples and quantitation of hPanx1 currents recorded from 1321N1 cells exposed to high K+ and BzATP in the presence of 100 μM carbenoxolone (CBX, a gap junction and Panx1 channel blocker). Note in E that CBX prevented the increase in hPanx1 currents induced by BzATP. The generation and maintenance of 1321N1 cells expressing the rP2X7 were previously described in [35]. (TIF) [file pone.0295710.s002.tif]
